# Beyond Viral Assembly: The Emerging Role of HIV-1 p17 in Vascular Inflammation and Endothelial Dysfunction

**DOI:** 10.3390/ijms262411949

**Published:** 2025-12-11

**Authors:** Ylenia Pastorello, Nicoleta Arnaut, Mihaela Straistă, Francesca Caccuri, Arnaldo Caruso, Mark Slevin

**Affiliations:** 1 Department of Anatomy and Embryology, George Emil Palade University of Medicine, Pharmacy, Science, and Technology of Târgu Mureş, 540139 Târgu Mureş, Romania; ylenia.pastorello@gmail.com; 2 Department of Medical Chemistry and Biochemistry, George Emil Palade University of Medicine, Pharmacy, Science, and Technology of Târgu Mureş, 540139 Târgu Mureş, Romania; arnaut.nicoleta99@gmail.com; 3 Doctoral School of Medicine and Pharmacy, George Emil Palade University of Medicine, Pharmacy, Science, and Technology of Târgu Mureş, 540139 Târgu Mureş, Romania; straistamihaela@gmail.com; 4 Section of Microbiology, Department of Molecular and Translational Medicine, University of Brescia, 25123 Brescia, Italy; francesca.caccuri@unibs.it (F.C.); arnaldo.caruso@unibs.it (A.C.); 5 Center for Advanced Medical and Pharmaceutical Research (CCAMF), George Emil Palade University of Medicine, Pharmacy, Science and Technology of Târgu Mureş, 540139 Târgu Mureș, Romania

**Keywords:** human immunodeficiency virus type 1 (HIV-1), p17, endothelial activation, vascular dysfunction, neuroinflammation

## Abstract

p17, the human immunodeficiency virus type 1 (HIV-1) matrix protein traditionally associated with viral assembly, has been recently investigated for its extracellular functions linked to vascular damage. This review examines the molecular and pathogenic signatures by which p17 and its variants (vp17s) contribute to endothelial activation, aberrant angiogenesis, and vascular inflammation, highlighting their relevance even under effective antiretroviral therapy (ART). Specifically, p17 exerts chemokine-like activities by binding to chemokine (C-X-C motif) receptor-1 and 2 (CXCR-1/2) on endothelial cells (ECs). This interaction triggers key signaling cascades, including the protein kinase B (Akt)-dependent extracellular signal-regulated kinase (ERK) pathway and endothelin-1/endothelin receptor B axis, driving EC motility, capillary formation, and lymphangiogenesis. Variants such as S75X demonstrate enhanced lymphangiogenic potency, associating them with tumorigenic processes involved in non-Hodgkin lymphoma (NHL) pathogenesis. Importantly, p17 promotes endothelial von Willebrand factor (vWF) storage and secretion, implicating a pro-coagulant state that may trigger the increased thromboembolic risks observed in HIV-positive patients. Furthermore, p17 crosses the blood–brain barrier (BBB) via CXCR-2-mediated pathways, contributing to neuroinflammation by activating microglia and astrocytes and amplifying monocyte chemoattractant protein-1 (MCP-1) levels, therefore playing a critical role in the development of HIV-associated neurocognitive disorders. Hence, the elaboration of potential therapeutic strategies finalized at inhibiting p17/vp17s’ interaction with their receptors could complement ART by addressing HIV-related neurovascular morbidity.

## 1. Introduction

The human immunodeficiency virus type 1 (HIV-1) represents the most common type of HIV, responsible for 95% of infections worldwide. HIV-1 belongs to the family of retroviruses, *Lentovirus genus*, exerting its pathogenic effect by targeting CD4 T cells, therefore impairing immunological functions [1,2]. Infection with HIV-1 has been extensively linked to enhanced risk of cardiovascular disease development, including myocardial infarction, heart failure, and pulmonary hypertension. The advent of prompt and effective antiretroviral therapy (ART) improved life expectancy but concomitantly enabled the increased incidence of aging-related cardiovascular comorbidities, further augmenting overall risk [3,4,5,6,7,8,9,10]. In the past decade, p17, the HIV-1 matrix protein critically involved in several stages of the retroviral life cycle, was investigated for its extracellular, cytokine-like activity associated with deregulation of immune and endothelial cell (EC) signaling. These aberrant processes have been deemed as leading factors of chronic immune activation, endothelial dysfunction, lymphoangiogenesis, pro-thrombotic states, as well as systemic and neuro-inflammatory sequelae in HIV-1 positive individuals, even under ART [11]. Furthermore, p17’s structural variants (vp17s) have been demonstrated to carry an enhanced pathogenic potential in regard to tumorigenesis, specifically in non-Hodgkin lymphoma (NHL) [12].

This review aims to identify the molecular signatures responsible for the vascular burden induced by p17 and its variants, highlighting their endothelial and neuroinflammatory effects. Moreover, we examine current and potential therapeutic schemes, offering insights into the involved mechanisms that could be exploited to modulate their noxious activities.

## 2. Human Immunodeficiency Virus Type 1 (HIV-1) p17 Biology and Secretion

Upon viral maturation, Pr55Gag, a precursor polyprotein encoded by the *Gag* gene in HIV-1, is cleaved by HIV-1 proteases into several proteins, including p17 (N-terminal region of Gag), which is primarily recognized as the matrix protein that forms a protective shell beneath the viral membrane of mature virions. Beyond simply providing structural stability, p17 is crucial for virion assembly, nuclear export, membrane targeting, and the spatial organization of viral components [13]. These roles highlight that p17 is not a passive element of viral architecture but instead a dynamic regulator actively involved in the HIV-1 life cycle. As a result of its functional versatility, p17 participates in processes independent of virion formation, particularly through its secretion into the extracellular environment. The release of p17 occurs through two main pathways. In the classical mechanism of viral assembly and budding, p17 is incorporated into mature viral particles and disseminated into the extracellular milieu during viral proliferation. In addition, p17 is secreted through non-classical mechanisms, including exosomal encapsulation, microvesicle shedding, and direct interaction with phosphatidylinositol (4,5)-bisphosphate at the plasma membrane. In this latter process, the protein undergoes cleavage by host aspartyl proteases, enabling its extracellular release in bioactive forms [13,14,15].

Adding to this regulatory complexity, p17 secretion is strongly influenced by host inflammatory responses. Pro-inflammatory stimuli, particularly through the activation of the nuclear factor-kappa B (NF-κB) pathway, intensify HIV-1 gene expression and promote Gag and p17 production. This inflammation-driven signaling not only increases intracellular levels of the protein but may also enhances its extracellular secretion, thereby linking host immune activation directly with viral protein dissemination [16]. Notably, p17 secretion persists even during successful ART, resulting in its detection in plasma, lymphatic tissues, and endothelium even when active viral replication is suppressed. This phenomenon is attributed to the aforementioned non-classical pathway involving the action of cellular aspartyl proteases, which enables p17 release in the absence of a traditional signal peptide. Such secretion includes the canonical full-length matrix protein and the C-terminal-truncated variants, both subtypes being capable of performing essential extracellular functions [14,17,18]. The persistence of p17 outside the context of productive infection underlines its functional independence and implies that the protein contributes to viral pathogenesis even when viremia is fully suppressed. This paradox highlights p17 as a potential effector of residual HIV-associated complications. Recent studies have focused on structural variants of p17, particularly insertions within its C-terminal region and mutations that give rise to conformationally destabilized proteins known as vp17s. These variants display altered immunological modulations, influencing disease progression and potentially contributing to HIV-related comorbidities [19,20,21,22].

Collectively, these findings indicate that p17, in both canonical and variant forms, persists in the extracellular milieu and retains biological activity despite ART, emphasizing the necessity of elucidating its extracellular functions.

## 3. Extracellular Activities of p17

Extracellularly p17 acts as a viral cytokine, deregulating the immune response and tissue homeostasis by numerous mechanisms involving interactions with leukocytes and ECs. p17 displays the ability to target T cells, NK cells, plasmacytoid dendritic cells as well as monocytes and influence their activation, differentiation status and proliferative capacity. This phenomenon depends on the interaction between the NH_2_-terminal region of p17 and the p17 receptor (p17R) on the cellular surface [23,24].

**Figure 1:**
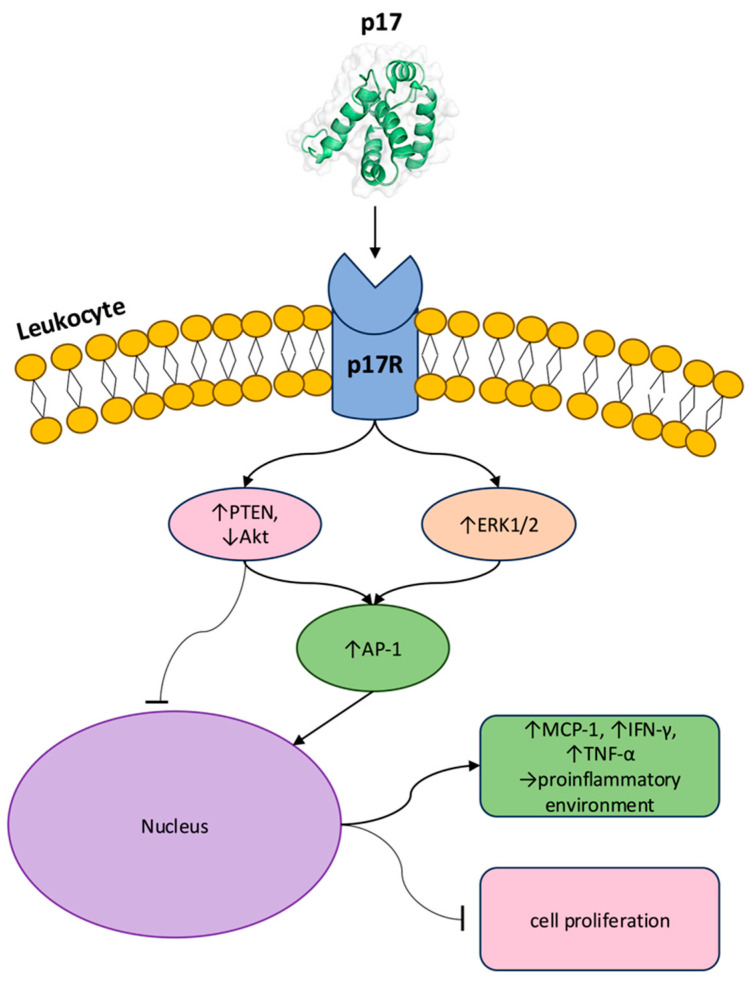
Molecular mechanisms induced by p17’s extracellular activity. The HIV-1 matrix protein p17 binds to its p17R on the surface of leukocytes, triggering multiple intracellular signaling cascades. This results in the activation of two principal pathways: upregulation (↑) of PTEN with subsequent Akt inhibition (↓), and upregulation of ERK1/2. Both events lead to the activation (↑) of the transcription factor AP-1. Enhanced AP-1 activity promotes nuclear responses leading to increased transcription (↑) of cytokines and chemokines, including MCP-1, IFN-γ, and TNF-α, establishing (→) a pro-inflammatory environment. Concomitantly, PTEN/Akt modulation inhibits cell proliferation. These effects collectively result in immune deregulation. Abbreviations: Akt, protein kinase B; AP-1, activator protein-1; ERK1/2, extracellular signal-regulated kinase 1/2; IFN-γ, interferon-gamma; MCP-1, monocyte chemoattractant protein-1; PTEN, phosphatase and tensin homolog; p17R, p17 receptor; TNF-α, tumor necrosis factor-alpha. Created with Microsoft PowerPoint.

Through p17-p17R binding, the protein rapidly activates the mitogen-activated protein kinase (MAPK)/extracellular signal-regulated kinase (ERK) pathway, as demonstrated by Giagulli et al. in an experiment involving Raji cells. Stimulation of 5 min with p17 and p17Δ36 (a 96 aa long truncated form) was found to analogously upregulate phosphorylation of ERK1/2 in Raji cells and consequently activate the transcription factor activator protein-1 (AP-1). Furthermore, the highly biologically active variant of p17, S75X, originating from a Ugandan HIV-1 strain (subtype A1), was tested at concentrations of 0.05, 0.1, 0.5 and 1 µg/mL, leading to an increased phosphorylation status of ERK1/2 at every selected dose [23]. This highlights p17 and vp17s’ role in modulating B cell survival, proliferation, and differentiation. In parallel, p17 modulates the phosphatase and tensin homolog (PTEN)/ phosphatidylinositol-3 kinase (PI3K)/ protein kinase B (Akt) pathway by suppressing the Akt pathway, via the activation of PTEN, a phosphatase which contrasts PI3K activity, therefore upregulating the cellular transcriptional factor AP-1. The COOH-terminal region of p17 mediates these processes through the serin/threonin (Ser/Thr) kinase ROCK, maintaining PTEN’s active form. PTEN activation and Akt inhibition regulate cell cycle progression and proliferation, indeed inducing the antiproliferative effect of p17 on B cells. Interestingly, the truncated forms, p17Δ36 and S75X, have been shown to trigger the activation of the PI3K/Akt signaling pathway. This is due to an inability to maintain PTEN in its active state, leading to increased PI3K/Akt activity which, in turn, promotes cell proliferation and malignant transformation [23,25]. At the transcriptional level, p17 engagement selectively drives AP-1 in primary human monocytes, which constitutively express p17Rs, further stimulating monocyte chemoattractant protein-1 (MCP-1) expression [26]. Functionally, p17 reinforces a pro-inflammatory milieu by potentiating interleukin-2 (IL-2)-driven immune activation. The work of De Francesco et al. proved that p17 induced freshly isolated peripheral blood mononuclear cells stimulated by IL-2 to increase release of tumor necrosis factor-alpha (TNF-α) and interferon gamma (IFN-γ), cytokines that have been shown to positively impact viral entry, reverse transcription, and proviral reactivation. In addition, interleukin-4 (IL-4) decreased IFN-γ and TNF-α levels, whereas p17 was discovered to restore the capacity of cells to synthesize both cytokines. Consequently, it could be speculated that the virus may employ p17’s to enhance pro-inflammatory cytokine production as a means to establish a more favorable environment for HIV-1 infection and replication [24] (Figure 1).

These findings highlight the complex effects of p17 and its variants on immune cell signaling, modulating key intracellular pathways and transcription factors that govern immune cell activation, proliferation, and sequelae, thus priming the vascular compartment for endothelial dysfunction, aberrant angiogenesis, and cumulative vascular damage.

## 4. Potential Mechanisms of p17-Induced Vascular Damage

### 4.1. Endothelial Activation and Proangiogenic and Lymphangiogenic Modulation

p17 exerts chemokine-like activity by binding to chemokine (C-X-C motif) receptor-1 (CXCR-1) on monocytes, mimicking interleukin-8 (IL-8) and eliciting monocytic adhesion as well as chemotaxis via Rho/Rho-associated protein kinase (ROCK) involvement. The protein also demonstrates high affinity for CXCR-2, an angiogenesis-mediating CXCR1-related cellular receptor [27,28]. Interaction with both CXCR-1 and CXCR-2, which are expressed on lymph node-derived lymphatic ECs (LN-LECs), activates the Akt-dependent ERK signaling pathway, resulting in capillary-like structure formation and enhanced LN-LEC motility [11,29]. Moreover, p17 has been shown to modulate lymphatic vessel formation via activation of the endothelin-1/endothelin receptor B axis [11]. The detection of p17 within the nucleus of ECs from an aviremic HIV-1 patient’s hepatic blood vessels further supports its broad impact on endothelial biology and supports its role as a direct regulator of cellular activities [11,29]. Experimental comparison of canonical p17 and its S75X variant in in vivo Matrigel models revealed that while both forms stimulate angiogenesis and lymphangiogenesis, S75X is notably more potent in promoting vascular structure formation, with increased patency and function. Additionally, S75X exposure resulted in pronounced adipocytic invasion, an outcome consistently linked to adipogenesis and, subsequently, angiogenesis [30,31,32]. Complementary studies in human brain endothelial cells (HbECs) found that p17 induces significant spheroid sprout formation and triggers chemotactic response, as demonstrated by polarized cellular directional movement when two adjacent spheroids are present. Furthermore, HBEC exposure to p17 for 8 min induced an upregulation of epidermal growth factor receptor (EGFR)-1-Y1172, ERK1/2, phospholipase C-gamma 1 (PLC-γ1), and focal adhesion kinase (FAK), all key intermediates in the angiogenic signal transduction activation and endothelial migration [33].

Consequently, p17 emerges as a pivotal mediator of abnormal vascular remodeling through its capacity to induce both angiogenic and lymphangiogenic responses. This established link to vascular dysfunction provides a critical foundation for examining the downstream consequences of endothelial activation and, specifically, how p17 further disrupts hemostatic balance and promotes pro-thrombotic states that aggravate the vascular pathology associated with HIV-1 infection.

### 4.2. Pro-Thrombotic Effects

HIV-1 infection is widely recognized for its association with coagulation abnormalities, heightened thrombotic risk, and increased predisposition for atherosclerosis development, primarily due to persistent inflammation and endothelial dysfunction [32]. Central to this process is p17, whose activation of ECs plays a pivotal role in aberrant vascular conditions. Specifically, in the study lead by Bugatti et al., p17 has been proven to promote von Willebrand factor (vWF) cytoplasmic accumulation in human umbilical vein ECs (HUVECs) and in a human lung microvascular ECs (HLMVECs) model [34]. In 2013, Torisu et al. established that autophagy regulates the processing, maturation, and secretion of vWF in ECs. When autophagic flux in ECs was disrupted, the release of vWF decreased, and prolonged bleeding time was observed in mice undergoing chloroquine treatment [35]. Provided that p17-mediated lymphangiogenesis is dependent on the endothelin-1/endothelin receptor B axis and the secretory autophagy pathway, it is relevant to investigate whether p17 can facilitate vWF storage and secretion from endothelium and the role reserved to autophagy in its activity. In this context, utilization of 3-MA, a synthetic and cell-permeable autophagic sequestration blocker, significantly diminished vWF cytoplasmic storage caused by p17 stimulation in HUVECs, which was further confirmed also in HLMVECs [11,34,36].

Hence, by promoting vascular dysfunction and accumulation of vWF within the ECs, p17 arises as a critical pro-coagulant factor, potentially contributing to the thromboembolic events associated with in HIV-1 infection. The lymphangiogenic activities of p17 not only drive abnormal vascular remodeling and activation but also contribute to the creation of a microenvironment that support tumor growth and persistence. Such predisposing milieu is particularly relevant as the persistent presence of p17 in lymphoid tissues has been associated with the development and progression of acquired immunodeficiency syndrome (AIDS)-related lymphomas [37].

### 4.3. Tumorigenic Effects

Despite the introduction of combined ART (cART), which substantially reduced the incidence of AIDS-defining cancers, NHL continues to be the most prevalent cancer-related comorbidity in HIV-1. Recent studies have highlighted the fundamental role of p17 and its variants in lymphomagenesis, irrespective of the patient’s immunodeficiency status [37]. Dolcetti et al. characterized the most prevalent vp17s in infected cellular reservoirs within lymphoma biopsies of five HIV-1-associated NHL patients; mutations were evidenced as amino acid insertions at position 117–118 (Ala–Ala) or 125–126 (Gly–Asn or Gly–Gln–Ala–Asn–Gln–Asn). Worth of notice is that all examined individuals presented low-level viremia [38]. Specifically, a vp17 sequence, p17-Lyrm, also displaying a 117 to 118 Ala-Ala insertion was identified in both plasma and splenic marginal zone lymphoma tissue of a HIV-1-related NHL patient in the study of Caccuri et al. [39]. As mentioned previously, vp17s and particularly S75X appear to be common and exert immunomodulatory activities that partially differ from the reference protein. In this context, activation of the Akt signaling and promotion of transformed B cells proliferation represent the most researched features [13,23,40].

While canonical p17 displays high affinity for CXCR-2, certain vp17s predominantly interact with the protease-activated receptor-1 (PAR-1) located on the surface of B cells. By activating PAR-1, “transactivation” of EGFR occurs via intermediary Gq proteins. EGFR activation stimulates the PI3K/Akt signaling pathway, whose activation is directly linked to B cell clonogenicity, a hallmark of lymphoma development. This vp17s-induced clonogenic process further requires matrix metalloproteinase (MMP) activity implicating these protein variants in both cellular expansion and tumor microenvironment remodeling [19,27,28]. Given that PAR-1 is widely expressed on ECs and its activation, especially by thrombin, leads to increased vascular permeability, loss of barrier integrity, and pro-thrombotic states, it is plausible to speculate a possible binding of v17p to PAR-1 on ECs, inducing further vascular dysfunction by activation of similar processes. This would complement the pro-lymphangiogenic modulation of v17p and the establishment of a microenvironment conducive to lymphoma proliferation and metastasis. However, to the best of our knowledge, no study has been conducted exploring this possibility.

**Figure 2:**
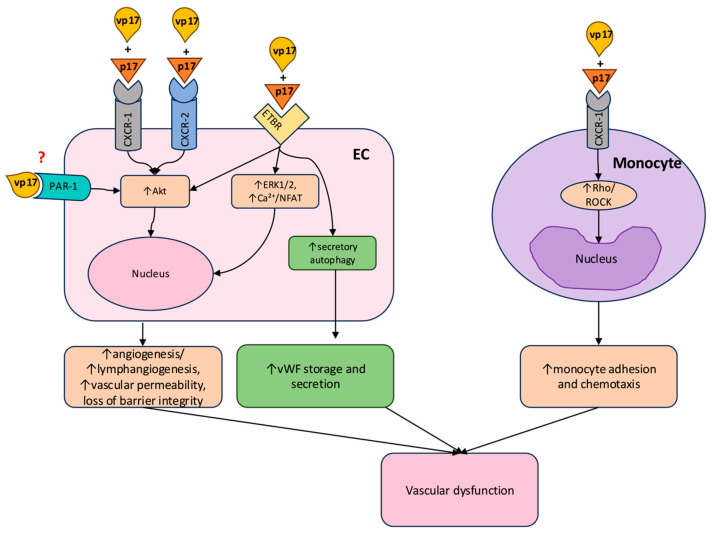
Mechanisms of p17/vp17-induced vascular dysfunction. In ECs, p17 and vp17s bind CXCR-1 and CXCR-2, as well as ETBR. Additionally, potential vp17’s binding to PAR-1 on ECs is contemplated. These interactions initiate Akt and ERK1/2 signaling cascades, increase (↑) intracellular Ca^2+^ and activate (↑) NFAT, collectively promoting (↑) angiogenesis, lymphangiogenesis, heightened vascular permeability, and loss of barrier integrity. Furthermore, activation of the endothelin-1/ETBR axis and secretory autophagy leads to increase (↑) in vWF storage and secretion, potentiating a pro-thrombotic endothelial state. In monocytes, p17/vp17 binding to CXCR-1 triggers Rho/ROCK-dependent signaling, resulting in enhanced (↑) monocyte adhesion and chemotaxis. The coordinated actions of p17/vp17s on ECs and monocytes create a pro-inflammatory and pro-coagulant microenvironment, leading to vascular dysfunction. This highlights their pivotal role in disrupting vascular and immune homeostasis, providing a molecular basis for HIV-associated comorbidities like atherosclerosis. Abbreviations: Akt, protein kinase B; Ca^2+^, calcium ion; CXCR-1/2, chemokine (C-X-C motif) receptor-1 and 2; EC, endothelial cell; ERK1/2, extracellular signal-regulated kinase 1/2; ETBR, endothelin B receptor; NFAT, nuclear factor of activated T-cells; PAR-1, protease-activated receptor 1; ROCK, Rho-associated protein kinase; vp17, variant of p17; vWF, von Willebrand factor. Created with Microsoft PowerPoint.

The processes involved in p17/vp17s-induced vascular dysfunction are summarized in Figure 2.

The multifaceted roles of p17 in vascular modulation and oncogenic signaling form the rationale for exploring how its signaling cascades extend beyond localized tissue effects to drive systemic inflammation, immune dysregulation, and enhanced disease progression in chronic HIV infection.

### 4.4. Systemic Pro-Inflammatory Signaling

HIV-1 p17 can be regarded as a classical deregulatory virokine that acts extracellularly to disrupt host immune homeostasis and sustain chronic systemic inflammation. Its primary mode of action involves the upregulation of pro-inflammatory cytokines such as TNF-α, IFN-γ, and MCP-1, as previously discussed [24,26]. Chronic immune activation is further validated in HIV-1 patients by the elevated expression of key systemic inflammation markers, including interleukin-6 (IL-6), C-reactive protein (CRP) and D-dimers, biomarkers closely linked to disease progression and comorbid risk. Their relevance is particularly distinct during the early stages of ART, when immune reconstitution inflammatory syndrome (IRIS) may occur. In this phase, monitoring these biomarkers could facilitate the identification of high-risk patient subgroups, enabling improved risk stratification and predicting future IRIS events, subsequent AIDS progression, and overall mortality [41,42,43,44]. Supporting this association, a nested case–control study (1:2 matching) reported that patients who developed AIDS-defining events or died within the first 12 months of ART showed markedly higher cytokine levels after one month of treatment. Specifically, TNF-α, IL-8, and IFN-γ concentrations were increased by 38%, 78%, and 97%, respectively, in these cases compared with controls. Moreover, patients with baseline CRP, D-dimer, and IL-6 values above the median exhibited a six-fold higher risk of experiencing AIDS events or death. Importantly, among those who developed IRIS, adjusted percent differences for D-dimer, CRP, and IL-6 remained significantly higher compared with non-IRIS subjects, evidencing a persistent inflammatory drive even after partial immune restoration [41]. Experimental studies provided mechanistic insight into these results. Namely, Martorelli et al. demonstrated that S75X significantly increased IL-6 production by primary Epstein–Barr virus (EBV)-infected B lymphocytes derived from unrelated donors, whereas p17 itself did not produce the same effect [40]. These findings support the thesis asserting that p17-related proteins can directly amplify IL-6 secretion, thereby stimulating broader downstream inflammatory cascades including hepatic stimulation of CRP synthesis, pro-coagulant activity via tissue factor upregulation, and endothelial activation leading to fibrinolysis and elevated D-dimer levels.

The capacity of p17 to perpetuate a systemic pro-inflammatory state, alongside the resulting chronic immune activation, contributes to the phenomenon known as “inflammaging” among HIV-positive individuals, where persistent low-grade inflammation accelerates biological aging and increases susceptibility to vascular and neurocognitive complications. Notably, pro-inflammatory mediators induced by p17, as well as p17 itself, can traverse the blood–brain barrier (BBB) and activate resident immune cells, directly contributing to neuroinflammatory processes [45]. This molecular crosstalk establishes a link between chronic HIV infection and central nervous system (CNS) dysfunction. Consequently, elucidating the specific neuroinflammatory signatures of p17 is crucial for comprehending the pathogenesis of HIV-associated neurocognitive disorders (HAND) and related neurological complications.

### 4.5. Neuroinflammatory Signatures

The HIV-1 matrix protein p17 exerts biological activity not only as a structural component of the virus but also as a bioactive molecule with significant implications in neuroinflammation and the pathogenesis of HAND. Its presence has been indeed confirmed in autoptic brain tissue of HIV-1 patients exhibiting severe AIDS encephalopathy, highlighting its direct involvement in CNS pathology [46]. This contribution arises through several interconnected mechanisms linking p17 and its variants to the onset and progression of neuroinflammation. One key mechanism proposed involves the ability of p17 to cross the BBB. Experiments using an in vitro BBB model, composed of immortalized murine brain ECs (bEnd.3), revealed that 100 nM Atto488-labeled p17 translocates across the barrier via a CXCR-2-dependent, autophagy-independent process. The translocation process is driven by CXCR-2, without involvement of CXCR-1, and includes endocytosis, intracellular vesicular trafficking, and exocytosis. Following its binding to CXCR-2, p17 is transported to early and recycling endosomes, as well as late endosomes and lysosomes. Complementary in vivo studies further confirmed its ability to penetrate the CNS after systemic injection of [125I]-p17 into the tail vein of healthy female mice, affirming its potential to directly influence brain tissues [45].

Within the CNS, p17 and its variants exert pro-inflammatory and cytotoxic effects. Immunohistochemical studies detected canonical p17 expression in main CNS immune cells, including microglia, mature macrophages, and multinucleated giant cells (MGC) of AIDS patients [47,48]. Moreover, p17-positively stained cortical astrocytes were localized in mice injected with the matrix protein [46]. The protein’s selective induction of the chemokine MCP-1, which is elevated in the cerebrospinal fluid (CSF) of AIDS patients with HAND and correlates with dementia severity, highlights its role in mediating monocyte migration into the CNS [26,49,50]. Furthermore, p17’s IL-8 mimetic activity, which is involved in HIV replication, potentiates this inflammatory cell recruitment and activation [27,51,52]. Collectively, these findings underline p17’s dynamic regulation of chronic immune and glial cell activation, thereby contributing to the development of a neurotoxic milieu.

At a structural level, p17 exhibits intrinsic amyloidogenic properties that align it with proteins implicated in neurodegenerative disorders like Alzheimer’s disease (AD). Nuclear magnetic resonance and X-ray crystallography studies have shown that p17 adopts a conformation consisting of five α-helixes and a highly basic β-strand platform, with its “coiled coil” motifs predisposing it to misfolding and aggregation [53,54,55]. Indeed, Zeinolabediny et al. in 2017 [46] identified p17 within the cerebral cortex and blood vessels of HIV-positive patients, frequently co-localizing with CD68-positive macrophages, β-amyloid (Aβ) plaques, and phosphorylated tau (p-tau). Intrahippocampal injection of 1 µL of p17 in mice further tested the effect of the matrix protein on behavioral and cognitive functions. p17 was found to induce neophobia, impaired exploratory behavior, and increased anxiety manifestations, as well as cognitive loss, decreased acquisition of spatial learning, and lack of object discrimination memory at 2–3 weeks after treatment. These results were supported by p17-positive staining in the hippocampal neurons and cortical microvessels of the injected animals, where p17 co-localized with CD105, a marker of angiogenesis and endothelial activation [46]. This vascular involvement of p17 is further validated by studies showing that stereotactic administration of p17 into the CA1 hippocampal region of mice results in significant vascular and cellular dysfunction: four months after injection, there was augmented expression of phosphorylated EGFR-1 with cytoplasmic staining in cortical and hippocampal neurons, situated proximally to the injection site. Notably, EGFR-1 co-localizes with p17 within microvessels of the cortex [33]. Aberrant signaling through EGFR-1 is a known driver of brain EC angiogenic activation and vascular dysfunction, both of which are characteristic features of AD. This dysfunctional EGFR-1 upregulation not only promotes pathological angiogenesis but also activates astrocytes and enhances Aβ42 peptide neurotoxicity, perpetuating neuroinflammation. The combined overexpression of EGFR and Aβ42 exerts a synergistic effect that exacerbates memory loss and cognitive decline [33,56,57].

**Figure 3:**
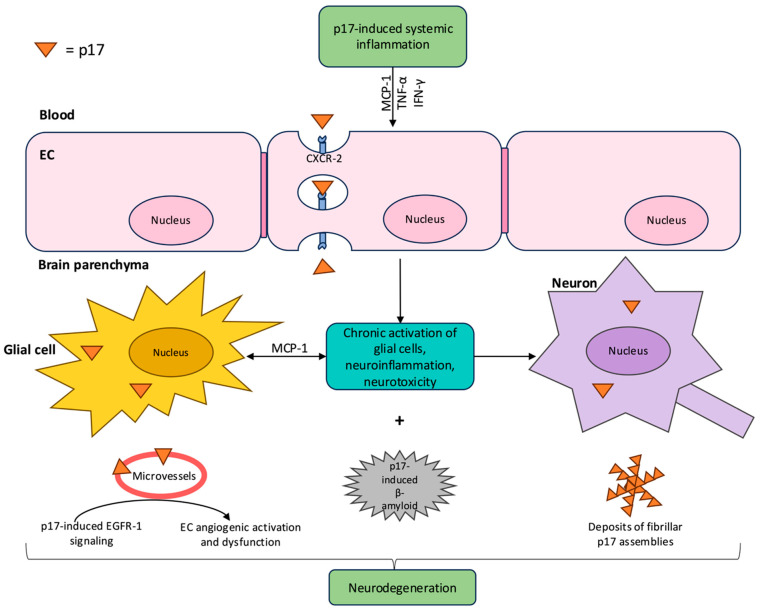
Systemic pro-inflammatory and neuroinflammatory signatures of p17. p17 drives systemic inflammation by inducing pro-inflammatory cytokines and chemokines-including MCP-1, TNF-α, and IFN-γ. Via CXCR-2–mediated translocation, p17 crosses the blood–brain barrier (BBB), accumulating in the brain parenchyma. Within the CNS, p17 localizes in glial cells, neurons, microvessels. Selective stimulation of MCP-1 leads to chronic activation of glial cells, with subsequent neuroinflammation and neurotoxicity. Additionally, this matrix protein enhances endothelial angiogenic signaling (via EGFR-1), disrupts EC function and vascular homeostasis. Remarkably, p17 also possesses amyloidogenic properties: it promotes the formation and deposition of fibrillar assemblies and β-amyloid, co-localizing with neurodegenerative hallmarks such as phosphorylated tau (p-tau). These aggregates are associated with behavioral deficits and cognitive impairment in animal models, paralleling Alzheimer’s disease-like pathology. The intricate actions of extracellular p17, including persistent neuroinflammation, pro-angiogenic dysfunction, and amyloidogenesis, converge to drive neurodegeneration and the development of HIV-associated neurocognitive disorders (HAND). Abbreviations: CXCR-2, chemokine (C-X-C motif) receptor-2; EC, endothelial cell; EGFR-1, epidermal growth factor receptor-1; IFN-γ, interferon-gamma; MCP-1, monocyte chemoattractant protein-1; TNF-α, tumor necrosis factor-alpha. Created with Microsoft PowerPoint.

Thus, the vascular dysfunction induced by p17 is inherently linked to its neurotoxic effects and provides a critical connection between viral protein activity, endothelial dysfunction, and the neurodegenerative processes observed in HAND and AD (Figure 3). This association emphasizes the importance of vascular pathology as a core feature of p17’s contribution to brain injury in long-standing HIV-1 infection and prompts further investigation into targeted therapies that disrupt these vascular and cellular interactions.

The respective roles of p17, S75X, and C-terminal insertions in chronic immune activation, vascular dysfunction, lymphangiogenesis, pro-thrombotic states, tumorigenesis, systemic inflammation, and neuroinflammatory processes are summarized and compared in Table 1.

## 5. Antiretroviral Therapy (ART) and Vascular Function in Chronic HIV-1 Infection

The advent of cART revolutionized the management of chronic HIV-1 infection. By suppressing viral replication and preserving immune function, cART has dramatically reduced morbidity and mortality among HIV-1-positive individuals. Typical cART regimens consist of at least two nucleoside reverse transcriptase inhibitors (NRTIs) paired with a third agent from a different class, such as an integrase inhibitor, non-nucleoside reverse transcriptase inhibitor (NNRTI), or protease inhibitor (PI), in order to reduce resistance risk and increase long-term efficacy [58,59,60,61,62,63]. Despite its undeniable value, the use of ART has been extensively associated with cardiovascular sequalae, exacerbating the already present predisposition of HIV-1 patients to develop such comorbidities [64,65,66]. The underlying mechanisms through which this occurs include decrease endothelial nitric oxide synthase (eNOS), increased expression of reactive oxygen species (ROS), cholesterol ester accumulation in macrophages, impaired cholesterol efflux from foam cells, as well as augmented proliferation of vascular smooth muscle cells (VSMCs) following insult [67]. This chronic inflammatory state is reflected in circulating levels of inflammatory biomarkers, particularly CRP. In this context, Guimarães et al. found significantly higher high-sensitivity CRP (hsCRP) levels in ART-treated patients compared to ART-naïve individuals (*p* < 0.001), with 56% of ART recipients exceeding the 3 mg/dL threshold indicative of high cardiovascular risk, versus 26% among the ART-naïve group. Importantly, hsCRP levels did not correlate with either CD4 cell counts or HIV viral load, highlighting the distinctive contribution of ART to this inflammatory profile [68]. These associations are further validated by a meta-analysis confirming increased hsCRP levels and marked heterogeneity in lipid profiles among treated patients. HIV-1 positive individuals on ART exhibited significantly higher LDL cholesterol, total cholesterol, and triglycerides than their non-receiving counterparts [69]. These findings confirm the necessity for routine lipid profile screening and cardiovascular risk assessment in HIV-1-infected individuals, particularly those undergoing ART [70]. Given the evidence pointing to ART as a significant modulator of vascular health, research has focused on the functional and structural vascular implications of its prolonged use. In a clinical trial, Borges et al. reported diminished cutaneous vascular conductance responses to acetylcholine and greater arterial stiffness in ART-treated patients compared to age-matched HIV-negative controls [71]. Furthermore, Lorenz et al. showed that chronic HIV infection and ART contribute to increased subclinical atherosclerosis, with common carotid intima-media thickness (IMT) and carotid bifurcation IMT raised by 5.7% and 24.4%, respectively, compared to controls (*p* < 0.0001). Notably, ART exposure for over two years was linked to a 19.7% greater carotid bifurcation IMT than in ART-naïve patients [72]. In addition, carotid arterial distensibility was found to be significantly lower among patients with HIV taking ART compared to non-users, data pointing to an augmented arterial stiffness driven by this therapeutic regimen [73]. Further distinctions and comparisons could be drawn between the various ART drug categories and their treatment durations; however, such analysis falls outside the scope of this review.

These detrimental vascular effects are complemented by studies showing that HIV-1 structural proteins and glycoproteins persist despite the absence of detectable viral replication under ART. Remarkably, immunohistochemical analyses revealed the presence of p17 in the germinal centers (GCs) of lymph nodes from seven patients affected by chronic HIV-1 infection, both before and after receiving treatment for a duration spanning between 5 and 13 months. The staining pattern for p17 did not differ between baseline tissue specimen and follow-up biopsy, suggesting continuous tissue presence regardless of effective viral suppression. Supporting this, serum detection of specific p17 antibodies by ELISA, before and after 5–13 months therapy regimen, revealed a circa 4-fold decrease in antibody titers against p17, pointing to a possible helper T cell-dependent generation of anti-HIV-1p17 antibodies [37]. These data are pertinent to the context of lymphangiogenic modulation mediated by vp17s during GC formation and B cell activation. Specifically, vp17s interact with mononuclear cells, thereby stimulating cytokines and chemokines that are essential for cell activation and proliferation. Long-standing accumulation of vp17s within the GC and its interaction with B cells in situ, suggests the persistence of a risk factor for NHL development not addressed by ART [37,74].

These findings demonstrate the multifaceted impact of ART and the persistent HIV-1 structural protein p17 on vascular integrity and lymphoproliferative risk, emphasizing the limitations of current therapeutic strategies in effectively managing HIV-associated comorbidities. Beyond p17, other HIV-1 proteins have been proven to contribute to the chronic inflammatory environment and related pathological complications.

## 6. Comparative Roles of Other HIV-1 Proteins on Endothelial Dysfunction and Neuroinflammation: Tat and Nef

Although HIV-1 cannot productively replicate within ECs, endothelial dysfunction arises from a combination of HIV-encoded proteins and host-derived inflammatory mediators secreted by infected immune cells. This interaction perpetuates vascular inflammation and contributes to the chronic endothelial injury observed in HIV-positive individuals. Beyond the matrix protein p17, other viral proteins such as the trans-activator of transcription (Tat) and the negative regulatory factor (Nef) have been strongly implicated in mediating endothelial impairment and promoting vascular complications associated with HIV infection [75,76,77,78].

### 6.1. Tat

Tat is encoded by the tat gene and classifies as a regulatory protein which drastically enhances the efficacy of viral transcription [79,80,81,82,83,84]. Its extracellular secretion relies on HIV-infected T-cells as well as monocytes and macrophages. Once in the circulation, Tat behaves as a proto-cytokine, modulating EC functions, among other cells [85]. Specifically, tat-induced endothelial dysfunction is instigated by dysregulation of ROS production and upregulation of interleukin-1 beta (IL-1β), MCP-1, vascular cell adhesion protein-1 (VCAM-1) and E-selectin expression through activation of the NF-κB signaling pathway, as demonstrated on HUVECs [75,86,87,88]. In this context, subcutaneous injection of Tat in mice induces a dose-dependent increase in vascular permeability, prompting lymphomononuclear cells recruitment, with the key involvement of MCP-1 and the platelet-activating factor (PAF) [89]. Supporting these findings, Kovacs et al., using aorta tissues of chronically Tat-treated mice, further corroborated that Tat stimulation results in the downregulation of eNOS and a concomitant reduction in nitric oxide (NO) bioavailability, effects mediated through induction of NADPH oxidase 1 (Nox1) and its co-activator NoxA1 [90]. The implications of Tat-related oxidative stress extend beyond vascular dysfunction. NADPH oxidase stimulation by Tat has been extensively linked to development of AIDS-related neurological manifestations as dementia and encephalitis [86]. Indeed, Song et al. showed that Tat upregulates expression of VCAM-1 and intercellular adhesion molecule-1 (ICAM-1) in activated microglia and astrocytes via a Nox-2-dependent mechanism, provoking pro-inflammatory responses within the CNS [91]. Correspondingly, studies on Tat-transgenic mice highlighted how this protein alters the BBB permeability and heightens microglial and perivascular macrophage activation in the dorsal striatum [92].

Hence, Tat’s dual capacity to induce endothelial injury and neuroinflammation through oxidative and inflammatory pathways establishes a crucial link between vascular dysfunction and HIV-related neurological complications.

### 6.2. Nef

Nef is an accessory HIV-1 protein that, despite being devoid of enzymatic activity, profoundly influences cellular signaling pathways and protein trafficking [93,94,95,96,97,98]. Besides enhancing viral infectivity and interfering with B-cell antibody maturation, Nef exerts potent pathogenic effects on the vascular endothelium [75]. It induces Nox2 complex activation and upregulation of ROS formation, leading to EC apoptosis, stimulation of ROS-sensitive pro-inflammatory pathway of NF-Kb, and finally instigation of MCP-1 production [86]. Wang et al. in 2014 confirmed the presence of Nef in ECs of CD4.Nef.GFP transgenic mice, demonstrating that Nef is transferred into the endothelium from infected T cells, in a Rac1-dependent fashion [99,100]. Experimental studies by Duffy et al. provided corroborative evidence using porcine pulmonary artery rings and cultured human pulmonary artery ECs (HPAECs) exposed to recombinant Nef. After 24 h of treatment, Nef significantly down-regulated eNOS expression, concomitantly increasing superoxide anion production in both specimens which is indicative of oxidative endothelial injury and impaired vasodilatory signaling, and decreased NO production in HPAECs by 21% compared with controls [101]. In addition to its vascular toxicity, Nef displays pronounced neurotoxic features. Implantation of Nef-expressing astrocytes into the rat hippocampus has been shown to increase BBB permeability, a hallmark and contributor to neuroinflammation, and induce impairments in both spatial and recognition memory [102,103]. Moreover, injection of Nef-containing extracellular vesicles (EVs) in the murine CNS decreased myelin basic protein (MBP) staining and reduced CC1+ oligodendrocytes counts within the corpus callosum, signifying disrupted myelination and direct impact on neuroinflammation [104].

Despite their potent cellular effects, Tat and Nef differ from p17 in terms of persistence and distribution. While Tat and Nef tend to exert more acute and pronounced impacts on cellular signaling and oxidative stress pathways, their presence in tissue generally diminishes after effective ART. In contrast, the ongoing secretion, extracellular stability, and tissue accumulation of p17 amplify its pathogenic influence, promoting chronic vascular alterations that develop insidiously over time. The persistence of p17 even under viral suppression suggests that targeting this protein may hold therapeutic potential for altering long-term vascular comorbidities in patients living with HIV.

## 7. Therapeutic Implications: Could Targeting p17 Reduce Vascular Comorbidities?

Counteracting p17’s noxious biological activities, as complementary measure to ART, represents a promising, comprehensive strategy aimed at addressing HIV-related complications and even AIDS. Fiorentini et al. explored the immunogenicity of p17-based peptides mimetic of the p17 epitope involved in p17R binding. Computational analysis revealed a 20 aa-long sequence (AT20) displaying significant mimicry with the functional loop present on the surface of p17. AT20 is characterized by a spatial conformation consisting of a partially unfolded α-helix, a feature retained among vp17s [105,106,107]. Pre-clinical testing on C57BL/6 mice immunized with the AT20 synthetic peptide, coupled to the carrier protein keyhole limpet hemocyanin (AT20-KLH), resulted in development of p17-neutralizing antibodies effective in inhibiting the interaction between p17 and its receptors [105]. Moreover, antibodies produced by Balb/c mice receiving AT20-KLH were proven to neutralize the activity of p17 deriving from different strains carrying a critical mutation within AT20, demonstrating broad effectiveness [106].

Clinically, AT20-KLH candidate vaccine has been assessed in a phase I trial in asymptomatic HIV-1-positive patients receiving ART. Subjects immunized with five doses developed high titers of anti-AT20 antibodies, which persisted for up to 2 years after the last immunization. Importantly, sera derived from vaccinated individuals displaced the binding between p17 and p17Rs, and this candidate vaccine was deemed safe and well tolerated both locally and systemically [108,109]. Such results support the feasibility of utilizing p17 immunogenic epitopes to neutralize p17’s pathogenicity and complement ART.

An alternative route for immunization was explored in the work of Verrier et al., which detected a region of sequence similarity between the epitopes of anti-p17 neutralizing antibodies and anti-gp41 neutralizing 2F5 antibody. Cross-reactivity between p17 and 2F5 was verified and subsequently enhanced via modifications of the p17 sequence. Classical and mutated p17 were then used to immunize rabbits, which produced neutralizing antibodies able to identify both p17 and gp41. By exploiting the principle of cross-reactivity, this study introduces an intriguing, potential strategy for designing multifunctional antigens derived from p17 aimed at eliciting both protective responses against Gag and neutralizing antibodies against gp41 as component of HIV-1 vaccine formulation [110]. Monoclonal antibodies targeting p17 have also yielded promising preclinical results. MBS-3, a mouse immunoglobulin G (IgG) anti p-17, was capable of entirely block p17 binding to Rajii cells, whereas another antibody, MK-18, demonstrated strong neutralizing activity [23]. Additionally, the experimental monoclonal antibody which targets PAR-1 and ATAP2 effectively halted vp17-induced PAR-1 signaling and the related downstream effects in B cells, reducing their abnormal clonogenic activity associated with lymphomagenesis [19]. These findings emphasize the therapeutic potential of antibody-mediated blockade of specific p17 interactions implicated in HIV pathogenesis.

In conclusion, disrupting the interaction between p17 and its key receptors, CXCR1 and CXCR2, as well as between vp17s and PAR-1, emerges as a clinically relevant strategy to restrain the pro-angiogenic and pro-lymphangiogenic effects promoted by p17 and its variants. Such interventions may mitigate the amplified cardiovascular and neurological risks associated with HIV-1 infection, complementing ART’s viral suppression by addressing residual protein-driven pathologies. This framework serves as the foundation for future therapeutic advancements designed to enhance long-term health outcomes for HIV-1-positive individuals.

## 8. Conclusions and Future Directions

HIV-1 p17, beyond its classical role as a matrix protein, behaves as a multifunctional viral factor with persistent extracellular secretion contributing, along with its variants, to chronic immune activation, vascular dysfunction, lymphangiogenesis, pro-thrombotic states, tumorigenesis, systemic inflammation, and neuroinflammatory processes. Suppression of viral replication by ART does not abrogate p17’s presence within tissues and circulation, thus allowing pro-inflammatory and pathogenic milieus to be continuously maintained.

Despite substantial advancements in the identification of p17 and its variants’ vascular activity, several gaps in knowledge persist and require further investigation. Specifically, future studies should address the precise molecular mechanisms underlying p17/vp17’s effects on ECs, the long-term consequences of such vascular changes in vivo, the variability of these effects across different HIV-1 clades and patient populations, and the potential interactions with other viral or host factors influencing endothelial function. Such investigations would be essential for elucidating the role of p17 in HIV-associated vascular pathology and for facilitating the development of effective, targeted therapeutic strategies.

## Figures and Tables

**Table 1:** Roles of p17, S75X, and C-terminal insertions in key pathological processes. Abbreviations: Akt, protein kinase B; BBB, blood–brain barrier; CRP, C-reactive protein; CXCR-1/2, chemokine (C-X-C motif) receptor-1/2; EBV, Epstein–Barr virus; EC, endothelial cell; EGFR, epidermal growth factor receptor; ERK, extracellular signal-regulated kinase 1; ETBR, endothelin B receptor; FAK, focal adhesion kinase; HIV, human immunodeficiency virus; HLMVEC, human lung microvascular endothelial cell; HUVEC, human umbilical vein endothelial cell; IFN-γ, interferon gamma; IL-6, interleukin-6; LN-LEC, lymph node-derived lymphatic endothelial cell; MAPK, mitogen-activated protein kinase; MCP-1, monocyte chemoattractant protein-1; NHL, non-Hodgkin lymphoma; p17R, p17 receptor; PAR-1, protease-activated receptor 1; PI3K, phosphatidylinositol-3 kinase; PTEN, phosphatase and tensin homolog; TNF-α, tumor necrosis factor-alpha; vWF, Von Willebrand factor.

Pathological Biological Activity	p17 (Canonical)	S75X Variant	C-terminal Insertions
Chronic Immune Activation	Triggers pro-inflammatory cytokines via p17R/MAPK/ERK in monocytes [24,26]	Enhances B-cell proliferation and IL-6 production in EBV-infected B cells via PI3K/Akt [23,25,40]	Promotes B-cell activation in germinal centers [38,39]
Vascular Dysfunction	Induces endothelial activation, EGFR/ERK/FAK signaling, and abnormal remodeling via CXCR-1/2 in brain and lymphatic ECs [11,29,33]	More potent angiogenesis in ECs and Matrigel models [30,33]	Potential PAR-1 binding on ECs speculated to prothrombotic states
Lymphangiogenesis	Promotes lymphatic vessel formation via CXCR1/2-Akt/ERK and endothelin-1/ETBR axis in LN-LECs [11]	Stronger in vivo lymphangiogenesis than p17; adipogenic invasion linked to vascular support [30]	Contributes to pro-lymphangiogenic tumor microenvironment in NHL [38]
Pro-thrombotic States	Promotes vWF accumulation and secretion in HUVECs/HLMVECs via secretory autophagy [34]	Not specifically reported	Not specifically reported; possible via PAR-1 on ECs
Tumorigenesis	Antiproliferative on B cells via PTEN activation/PI3K/Akt inhibition; supports tumor microenvironment by increased angiogenesis/lymphangiogenesis [23]	Strong B-cell clonogenicity via PI3K/Akt activation, PTEN inactivation; lymphoma promotion [23,38]	Confers B-cell growth via PAR-1-EGFR-PI3K/Akt; prevalent in NHL [38]
Systemic Inflammation	Sustains IL-6, TNF-α, IFN-γ production; contributes to HIV-associated IL-6, CRP, D-dimer elevation and inflammaging [24,41]	Amplifies IL-6 and broader cascades in B cells [40]	Indirect, via chronic B-cell dysregulation [38]
Neuroinflammatory Processes	Crosses BBB via CXCR2; induces MCP-1, amyloidogenic aggregates, EGFR in neurons and microvessels; cognitive deficits [33,45,46]	More potent angiogenesis in ECs and Matrigel models [30,33]. Potential link to vascular dementia	Not specifically reported

## Data Availability

No new data were created or analyzed in this study. Data sharing is not applicable to this article.

## Abbreviations

The following abbreviations are used in this manuscript:

AD	Alzheimer’s disease
AIDS	Acquired immunodeficiency syndrome
Akt	Protein kinase B
AP-1	Activator protein-1
ART	Antiretroviral therapy
Aβ	β-amyloid
BBB	Blood–brain barrier
cART	Combined antiretroviral therapy
CNS	Central nervous system
CRP	C-reactive protein
CSF	Cerebrospinal fluid
CXCR-1/2	Chemokine (C-X-C motif) receptor-1/2
EBV	Epstein–Barr virus
EC	Endothelial cell
EGFR	Epidermal growth factor receptor
eNOS	Endothelial nitric oxide synthase
ERK	Extracellular signal-regulated kinase
EV	Extracellular vesicles
FAK	Focal adhesion kinase
GC	Germinal center
HAND	HIV-associated neurocognitive disorders
HbEC	Human brain endothelial cell
HIV-1	Human immunodeficiency virus type 1
HLMVEC	Human lung microvascular endothelial cell
HPAEC	Human pulmonary artery endothelial cell
hsCRP	High-sensitivity CRP
HUVEC	Human umbilical vein endothelial cell
ICAM-1	Intercellular adhesion molecule-
IFN-γ	Interferon gamma
IgG	Immunoglobulin G
IL-1β	Interleukin-1 beta
IL-2	Interleukin-2
IL-4	Interleukin-4
IL-6	Interleukin-6
IMT	Intima-media thickness
IRIS	Immune reconstitution inflammatory syndrome
LN-LEC	Lymph node-derived lymphatic endothelial cell
MAPK	Mitogen-activated protein kinase
MBP	Myelin basic protein
MCP-1	Monocyte chemoattractant protein-1
MGC	Multinucleated giant cells
MMP	Matrix metalloproteinase
Nef	Negative regulatory factor
NF-κB	Nuclear factor-kappa B
NHL	Non-Hodgkin lymphoma
NNRTI	Non-nucleoside reverse transcriptase inhibitor
NO	Nitric oxide
Nox-1	NADPH oxidase 1
NRTI	Nucleoside reverse transcriptase inhibitors
p17R	p17 receptor
PAF	Platelet-activating factor
PAR-1	Protease-activated receptor 1
PI	Protease inhibitor
PI3K	Phosphatidylinositol-3 kinase
PLC-γ1	Phospholipase C-gamma 1
PTEN	Phosphatase and tensin homolog
p-tau	Phosphorylated tau
ROCK	Rho-associated protein kinase
ROS	Reactive oxygen species
Ser/Thr	Serin/Threonin
Tat	Trans-activator of transcription
TNF-α	Tumor necrosis factor-alpha
VCAM-1	Vascular cell adhesion protein-1
vp17	p17 variants
VSMC	Vascular smooth muscle cells
vWF	Von Willebrand factor
